# Life on Mars, can we detect it?

**DOI:** 10.1038/s41467-023-36176-x

**Published:** 2023-02-21

**Authors:** Carol R. Stoker

**Affiliations:** NASA Ames Research Center, Space Science Division, MS 245-3, Moffett Field, California, CA 94035 USA

**Keywords:** Astrobiology, Biochemistry

## Abstract

Searching for evidence of life on Mars is a major impetus for exploration. A new study published in *Nature Communications* finds that current Mars mission instruments lack the essential sensitivity to identify life traces in Chilean desert samples that strongly resemble the martian area currently under study by NASA’s Perseverance rover.

Almost half a century ago the NASA Viking landers searched for evidence of life in Mars soils by attempting to detect active metabolism and measuring organic compounds by heating to vaporize them for detection via Gas Chromatography Mass Spectrometry (GC-MS). When no organic compounds were detected even at levels of parts per billion^1^, this provided a puzzle. Since organic compounds are continuously delivered to Mars by meteorites, comets, and interplanetary dust particles they should be ubiquitous and comprise up to 2% of Mars soil^2^. But where are they? This curious lack of organics argued both *against* a biological interpretation for results of the metabolism experiments, and *for* the presence of strong soil oxidants that actively destroy these compounds^3^. However, decades later the Viking GC-MS results were explained by the presence of high levels of perchlorate salts in the Martian soil^4^ that oxidized organics during pyrolysis in the Viking GC-MS instrument^5^.

While Viking lander results seemed unpromising for surface life in the cold, dry martian conditions, data from Viking orbiters and later missions showed that Mars had surface liquid water early in its history (3 to 4 billion years ago)^6^ where life may have thrived. This led later NASA rover missions to seek evidence of ancient habitable environments hosting organic remnants of life. The SAM (*Sample Analysis on Mars*) instrument on the Curiosity rover detected organics in ancient lakebed deposits^7,8^, but they are found at low levels (parts per billion) and appear to be highly altered. The Perseverance rover, now exploring an ancient illuvial fan river delta, has detected aromatic organic compounds using fluorescence spectroscopy^9^. But in these cases, the simple organics detected could have been delivered to Mars from space so proving a biological origin is impossible.

The paper by Azua-Bustos et al.^10^ in *Nature Communications* describes analysis of samples collected from “Red Stone”, an alluvial fan river delta more than 100 million years old in the Atacama Desert of Chile, Earth’s oldest and driest desert. Red Stone has similar geology to the delta area currently being studied by NASA’s Perseverance rover^11^ (Fig. 1). The Red Stone location (near the ocean) frequently experiences fogs that provide water for sparse but active microbial life that was detected by DNA extraction and gene sequencing, microscopy, and growth of a few microbial strains cultured from the samples. Most microbes identified consist of “microbial dark matter” i.e., genetic information is from organisms that have not yet been described.

**Fig. 1:**
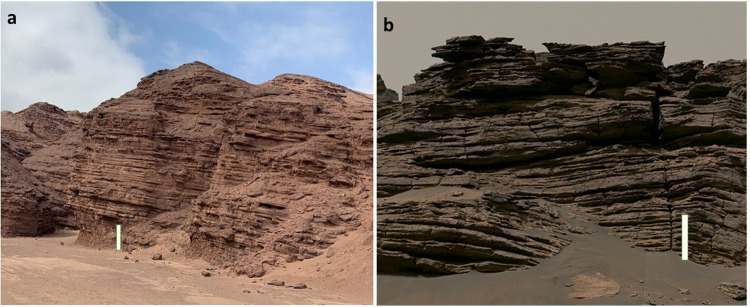
Comparison of the morphology of the Red Stone delta deposits with similar morphology of the delta deposits in Jezero Crater, Mars. **a** Red Stone deposit from panorama image courtesy of Armando Azua-Bustos. **b** A section of the panoramic composite image [image PIA24921_MAIN-20k.jpg] of the Jezero delta captured by the MastCam-Z camera on the Perseverance Rover in June 2022^15^ (Courtesy NASA/JPL-Caltech/ASU/MSSS). The height of both white scale bars are ~2 m.

Samples from Red Stone were also analyzed by instruments that simulate those used on Mars, but even finding organics in these samples was challenging. A GC-MS instrument comparable to but ten times more sensitive than Curiosity rover’s SAM instrument was barely able to detect specific biogenic organic compounds at the limit of detection, suggesting that SAM could not have detected them. There was more success with organic detection after sample extraction with a powerful solvent that was also implemented on the SAM instrument. Indeed, the SAM instrument detected organics in both sedimentary mudstones and dune sands on Mars by using solvent extraction and derivatization (a chemical treatment to make organic compounds easier to detect)^8^.

Red Stone samples were analyzed with a testbed of the MOMA (Mars Organic Molecular Analysis) instrument^12^ planned for the European ExoMars rover payload. No organics were detected when flash pyrolysis was used, but a few organic molecules were identified in evaporite samples when they were first extracted with a solvent and derivatized.

Red Stone samples were also analyzed with the SOLID-LDChip (Signs of Life Detector-Life Detector Chip)^13^, an instrument based on the technology of immunoassay that is commonly used in biomedicine. SOLID was designed for life detection on Mars but no scheduled missions plan to use it. Interestingly, the LDChip detected evidence of cyanobacteria that were not seen in the modern microbiota so Azua-Bustos et al.^10^ posit these biosignatures were deposited along with the delta sediments at least 100 million years ago.

This Red Stone sample analysis^10^ shows how critical it is to test instruments designed for life detection on other planets by using samples from relevant Earth analogs prior to selecting them for flight missions. If the biosignatures can’t be detected in Earth samples, where both current and ancient life is clearly documented, we should not expect these instruments to be capable of detecting evidence of life from Mars’ early history.

Azua-Bustos et al.^10^ argue that detection of ancient life signatures will require sample analysis in sophisticated terrestrial laboratories. This same view has led to the current plans for searching for life: the Perseverance rover is collecting samples that are to be retrieved and brought to Earth by a future mission^14^. But any biological activity in these samples presumably took place billions of years ago, and only a few small samples can be brought to Earth for study. It remains to be seen if unambiguous signatures of life can be found in those limited samples. We must be cautious about interpreting absence of strong evidence of life as evidence of its absence!
